# Correction: Investigation of the mechanism of the anomalous Hall effects in Cr_2_Te_3_/(BiSb)_2_(TeSe)_3_ heterostructure

**DOI:** 10.1186/s40580-023-00360-y

**Published:** 2023-02-20

**Authors:** Seong Won Cho, In Hak Lee, Youngwoong Lee, Sangheon Kim, Yeong Gwang Khim, Seung-Young Park, Younghun Jo, Junwoo Choi, Seungwu Han, Young Jun Chang, Suyoun Lee

**Affiliations:** 1 Center for Neuromorphic Engineering, Korea Institute of Science and Technology, Seoul, 02792 Korea; 2 Department of Materials Science and Engineering, Seoul National University, Seoul, 08826 Korea; 3 Center for Spintronics, Korea Institute of Science and Technology, Seoul, 02792 Korea; 4 Department of Physics, Konkuk University, Seoul, 05029 Korea; 5 Department of Materials Science and Engineering, Korea University, Seoul, 02841 Korea; 6 Department of Physics, University of Seoul, Seoul, 02504 Korea; 7 Department of Smart Cities, University of Seoul, Seoul, 02504 Korea; 8 Center for Scientific Instrumentation, Korea Basic Science Institute, Daejeon, 34133 Korea; 9 Division of Nano & Information Technology, Korea University of Science and Technology, Daejeon, 34316 Korea

**Correction: Nano Convergence (2023) 10:2** 10.1186/s40580-022-00348-0

Following publication of the original article [1], the author noticed an error in the corresponding authorship of the article. The typesetter has inadvertently missed to include co-corresponding authorship to Young Jun Chang at the time of correction process. This has been corrected with this erratum.
