# Supplementary material for: Investigation of the mechanism of the anomalous Hall effects in Cr2Te3/(BiSb)2(TeSe)3 heterostructure
Source: Nano Converg. 2023 Jan 10;10:2. doi: 10.1186/s40580-022-00348-0 (PMC9832196; doi:10.1186/s40580-022-00348-0)
Supplement: Supplementary file 1 — Additional file 1: Figure S1. TEM cross-section image of CT/BSTS. Figure S2. Schematics of the electrical measurement Figure S3. Magnetoresistance vs. H curves of BSTS, CT, and CT/BSTS. Figure S4. Scheme of decomposing the Hall resistivity. Figure S5. Separation of two AHE components at 50 – 90 K. [file 40580_2022_348_MOESM1_ESM.docx]

Additonal file Information for

Investigation of the mechanism of the anomalous Hall effects in Cr_2_Te_3_/(BiSb)_2_(TeSe)_3_ heterostructure

Seong Won Cho^1,2,†^, In Hak Lee^3†^, Youngwoong Lee^1,4^, Sangheon Kim^1,5^, Seungwu Han^2^, Young Jun Chang^6,7^, Suyoun Lee^1,8,^*

^1^Center for Neuromorphic engineering, Korea Institute of Science and Technology, Seoul 02792, Korea

^2^Department of Materials Science and Engineering, Seoul National University, Seoul 08826, Korea

^3^Center for Spintronics, Korea Institute of Science and Technology, Seoul 02792, Korea

^4^Department of Physics, Konkuk University, Seoul 05029, Korea

^5^Department of Materials Science and Engineering, Korea University, Seoul 02841, Korea

^6^Department of Physics, University of Seoul, Seoul 02504. Korea

^7^Department of Smart Cities, University of Seoul, Seoul 02504, Korea

^8^Division of Nano & Information Technology, Korea University of Science and Technology, Daejeon 34316, Korea

^†^ These authors contributed equally.

* e-mail: slee_eels@ kist.re.kr

**
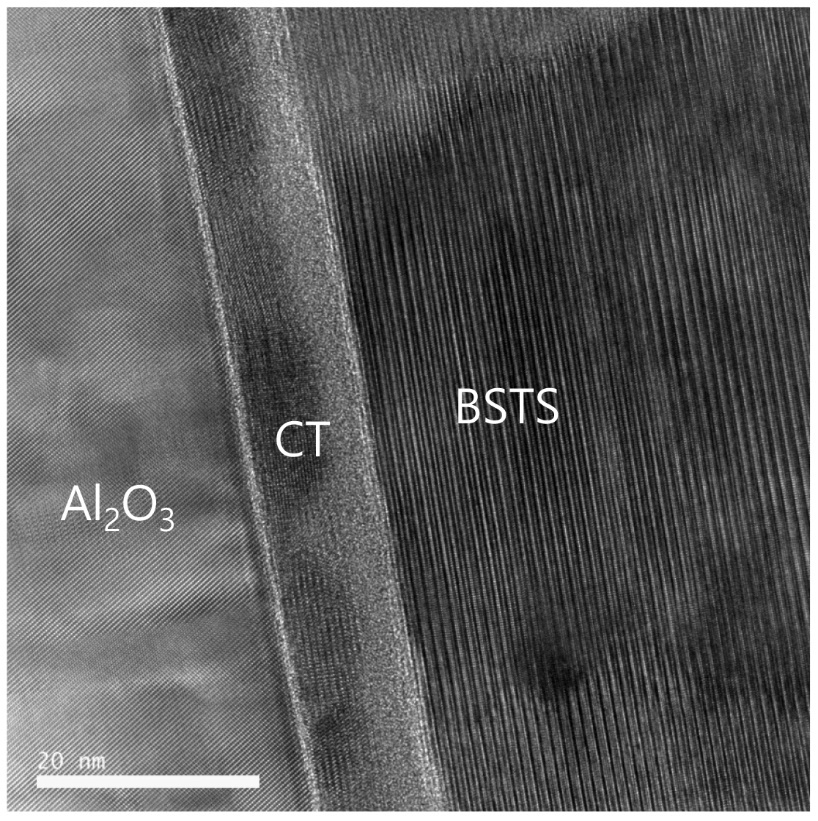
**

**Fig. S1. TEM cross-sectional image of CT / BSTS heterostructure**.

**
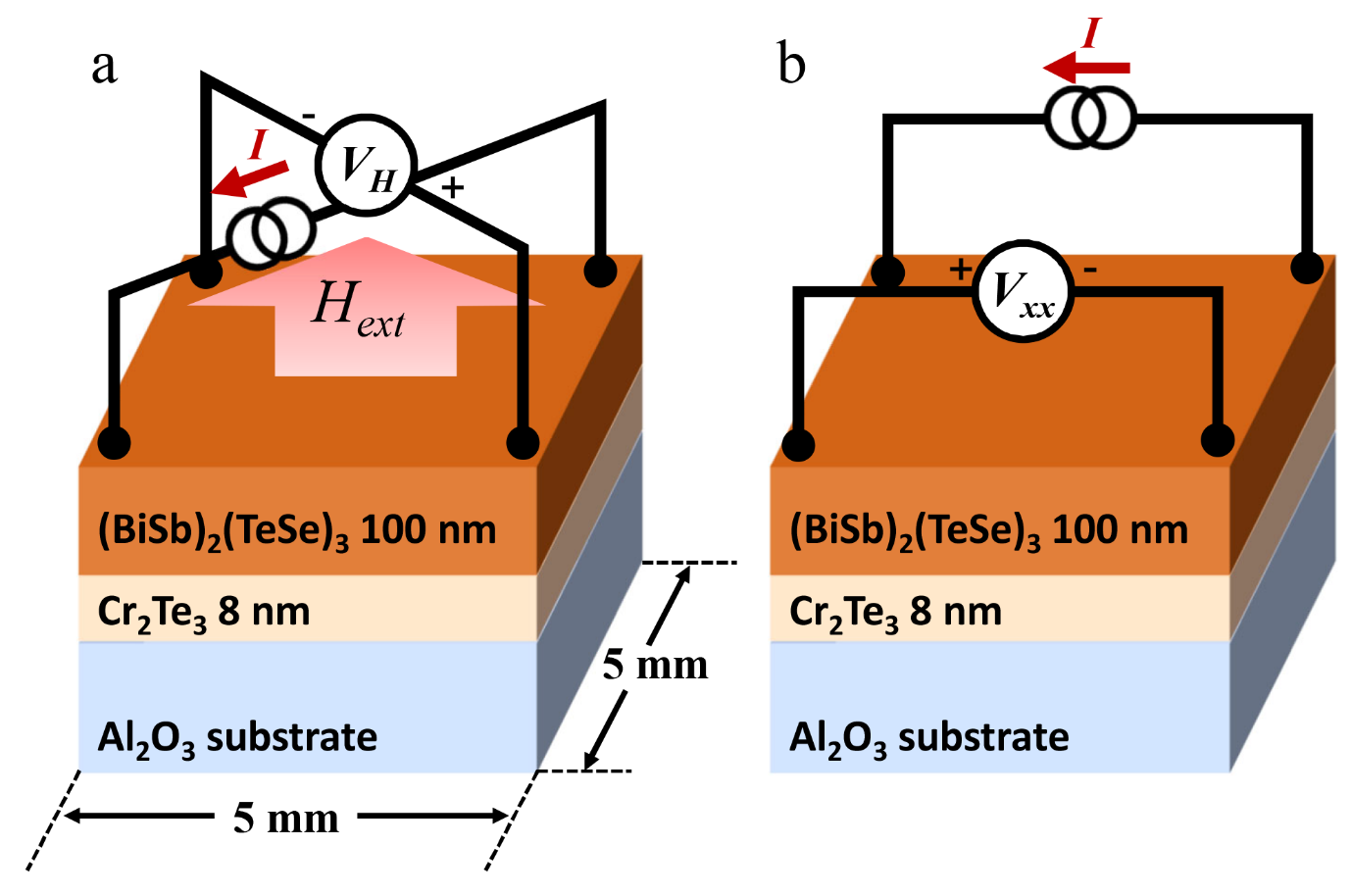
**

**Fig. S2****. Schematic illustration of the CT / BSTS heterostructure and the measurement setup.** The Hall (a) and longitudinal resistance (b) are measured using the conventional Van der Pauw (VdP) method with samples of 5 mm × 5 mm square.

**
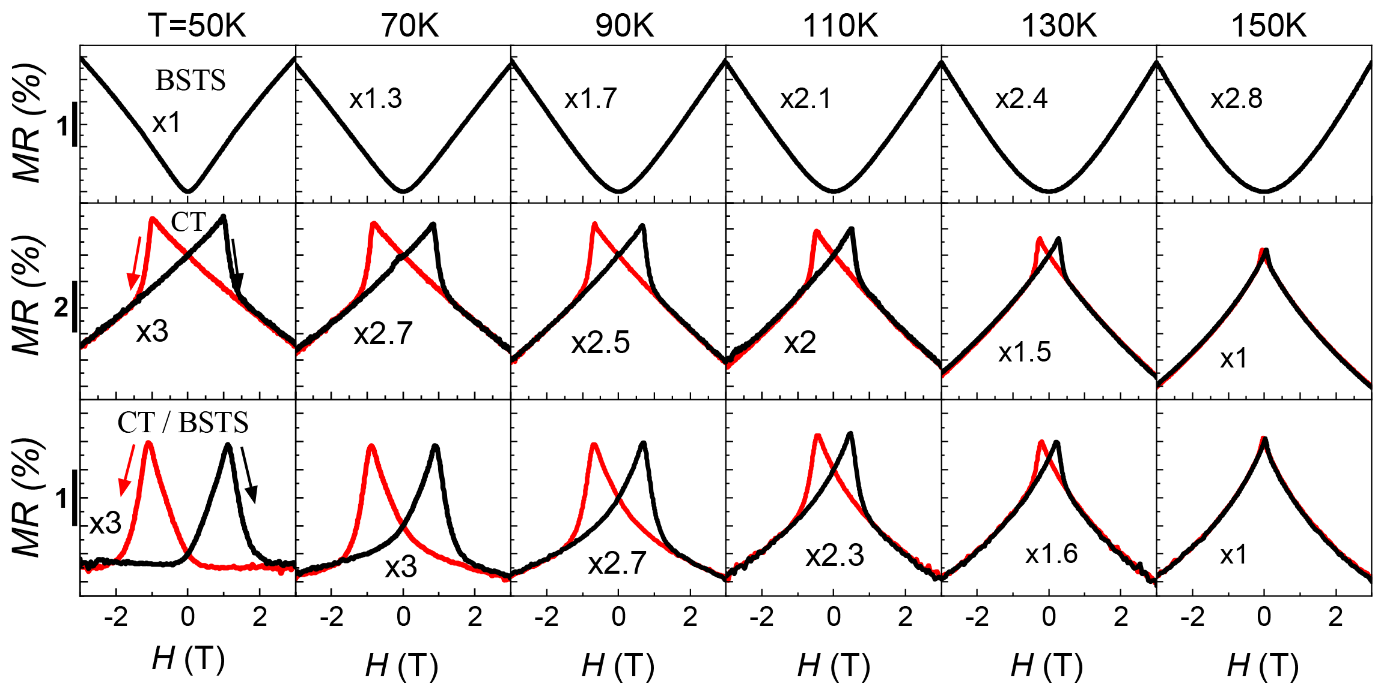
**

**Fig. S3. The magnetoresistance vs. magnetic field curves of BSTS, CT, and CT/BSTS heterostructure.** The measurement temperature is indicated at the top of the panel. The black and red curves represent the cases for the ascending and descending magnetic fields, respectively.


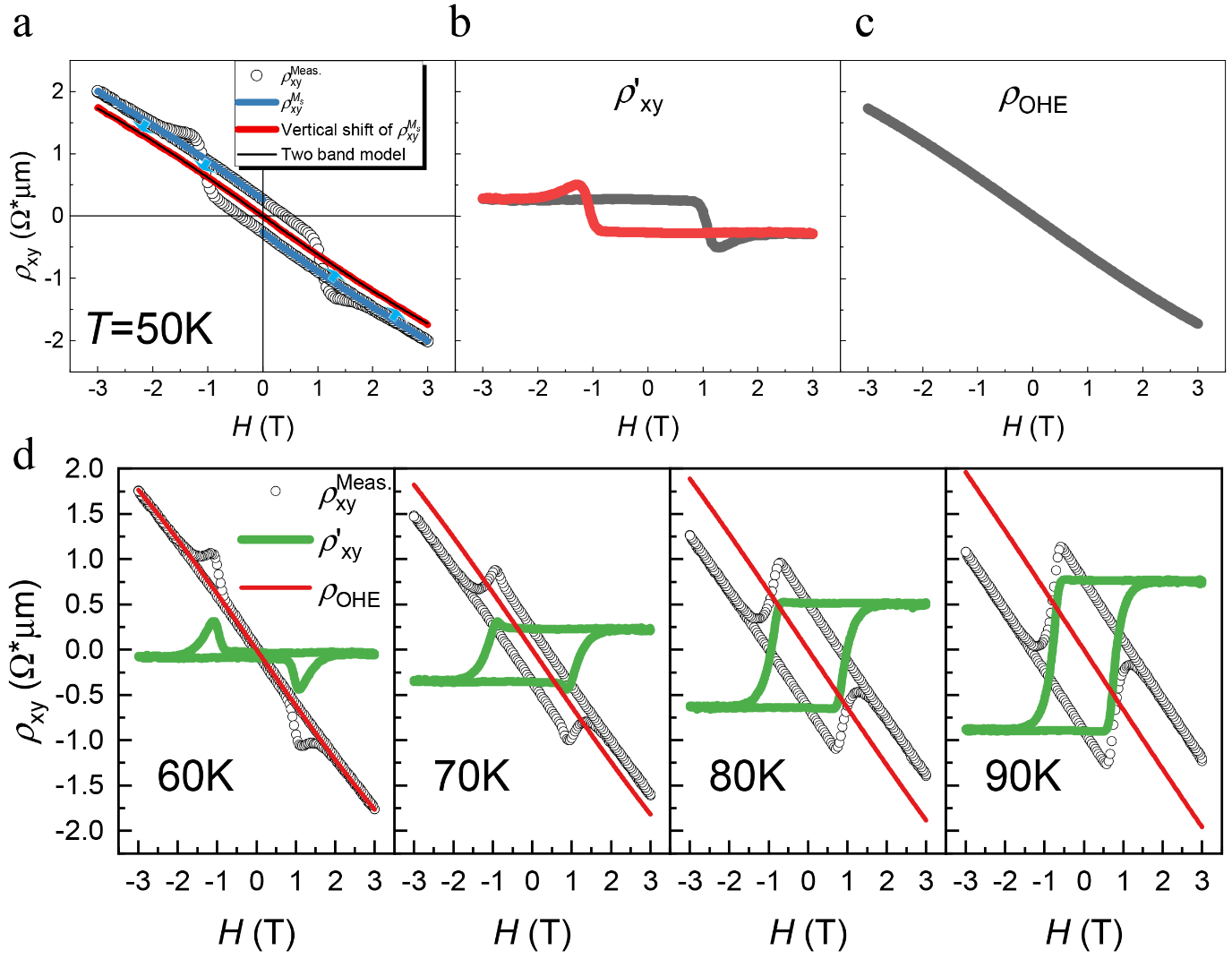


**Fig. S4. Process of decomposing the Hall resistivity.** The process of decomposing the Hall resistivity at 50K is represented in a, and the process is summarized as follows:

Step 1> Extract $\rho_{xy}^{M_{s}}$ from $\rho_{\mathrm{xy}}^{meas.}$ where the magnetization is saturated and far enough away from the coercive field (blue line).

Step 2> Add an offset so that the extracted $\rho_{xy}^{M_{s}}$ crosses the origin (red line).

Step 3> The red line obtained through the above process represents the ordinary Hall effect. Fit this curve through the two-band model which expresses the nonlinear ordinary Hall effect (black line).

Step 4> The reduced Hall resistivity ($\rho_{\mathrm{xy}}^{'}=\rho_{\mathrm{AHE}}+\rho_{\mathrm{hump}}$) is extracted by subtracting the ordinary Hall resistivity ($\rho_{\mathrm{OHE}}$) from $\rho_{\mathrm{xy}}^{meas.}$.

The extracted $\rho_{xy}^{'}$ and $\rho_{OHE}$ from the above process are shown in b and c, respectively. The decomposition results of $\rho_{xy}^{meas.}$ at 60-90K are shown in d.

**ss
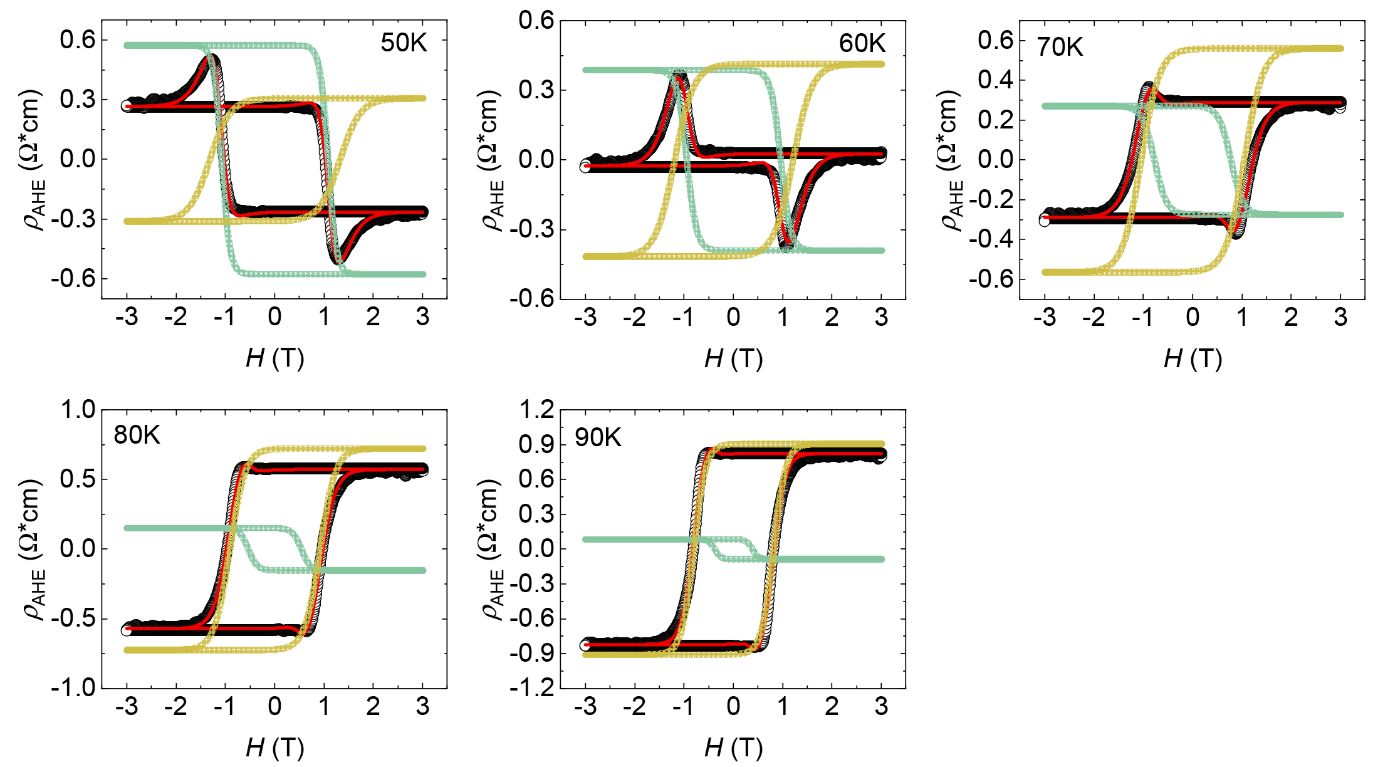
**

**Fig. S5. Separation of two AHE components by the mathematical fitting.** The black open circle indicates the raw data. The yellow and green lines represent the component giving the positive and negative AHE, respectively. The red curve is the sum of those two components.
